# Pharmacokinetic Analysis of [^18^F]FES PET in the Human Brain and Pituitary Gland

**DOI:** 10.1007/s11307-023-01880-z

**Published:** 2024-01-23

**Authors:** Nafiseh Ghazanfari, Janine Doorduin, Chris W. J. van der Weijden, Antoon T. M. Willemsen, Andor W. J. M. Glaudemans, Aren van Waarde, Rudi A. J. O. Dierckx, Erik F. J. de Vries

**Affiliations:** Department of Nuclear Medicine and Molecular Imaging, University of Groningen, University Medical Center Groningen, Hanzeplein 1, 9713GZ Groningen, The Netherlands

**Keywords:** Estrogen receptor, Receptor density, Positron emission tomography, Neuroimaging, Kinetic modeling

## Abstract

**Purpose:**

Estrogen receptors (ER) are implicated in psychiatric disorders. We assessed if ER availability in the human brain could be quantified using 16α-[^18^F]-fluoro-17β-estradiol ([^18^F]FES) positron emission tomography (PET).

**Procedures:**

Seven post‑menopausal women underwent a dynamic [^18^F]FES PET scan with arterial blood sampling. A T1-weighted MRI was acquired for anatomical information. After one week, four subjects received a selective ER degrader (SERD), four hours before the PET scan. Pharmacokinetic analysis was performed using a metabolite-corrected plasma curve as the input function. The optimal kinetic model was selected based on the Akaike information criterion and standard error of estimated parameters. Accuracy of Logan graphical analysis and standardized uptake value (SUV) was determined via correlational analyses.

**Results:**

The reversible two-tissue compartment model (2T4k) model with fixed K_1_/k_2_ was preferred. The total volume of distribution (V_T_) could be more reliably estimated than the binding potential (BP_ND_). A high correlation of V_T_ with Logan graphical analysis was observed, but only a moderate correlation with SUV. SERD administration resulted in a reduced V_T_ in the pituitary gland, but not in other regions.

**Conclusions:**

The optimal quantification method for [^18^F]FES was the 2T4k with fixed K_1_/k_2_ or Logan graphical analysis, but specific binding was only observed in the pituitary gland.

**Supplementary Information:**

The online version contains supplementary material available at 10.1007/s11307-023-01880-z.

## Introduction

Estrogen receptors (ER) are associated with regulatory functions in the central nervous system (CNS) and exert neuroprotective and neurotrophic effects [1]. Substantial evidence suggests that estrogens play an important role in psychiatric disorders, such as postmenopausal and postnatal depression [2]. Hormone replacement therapy can improve such postmenopausal complaints [3]. Although the ER is supposedly involved, there is not much known about its exact role in these psychiatric disorders. Estrogen receptor density in the living human brain may be assessed by non-invasive imaging techniques, such as positron emission tomography (PET). Several PET tracers have been developed for the purpose of ER imaging [4]. Among these, 16α-[^18^F]-fluoro-17β-estradiol ([^18^F]FES) is the best-characterized tracer with high affinity and selectivity for ER. [^18^F]FES PET is now used in clinical trials and regular patient care for imaging of ER expression in hormone-sensitive tumors, mainly breast cancer [5, 6], but may also be suitable for ER imaging in the brain. The first study using [^18^F]FES PET to assess ER expression in rat brains found the highest [^18^F]FES uptake in pituitary gland and hypothalamus, regions with high ER expression. Co-administration of 17β-estradiol with the tracer led to a decrease in tracer uptake in those two brain regions [7], suggesting that tracer uptake was ER-mediated. Despite these promising preclinical results, [^18^F]FES PET has not been fully evaluated for imaging of ER expression in the human brain yet. Whole-body PET images of cancer patients show heterogenous uptake (SUV) of the tracer in the brain, with higher uptake in white than grey matter [8, 9], but it is unclear whether this uptake is ER-mediated. The purpose of the current study was to determine if [^18^F]FES PET can be used for imaging of ER expression in the human brain and to assess what the optimal method is to quantify [^18^F]FES binding. The selective ER degrader elacestrant was administered to assess whether [^18^F]FES uptake in the brain is ER-mediated.

## Materials and Methods

### Experimental Design and Study Set-up

PET imaging was carried out as part of a phase 1 study of the experimental drug elacestrant, which aimed to determine ER availability [10]. Elacestrant was developed as a selective estrogen receptor degrader that crosses the blood–brain barrier for the treatment of estrogen receptor positive breast cancer brain metastases. The drug was shown to competitively bind to ERs [11] in the same binding pocket as estrogen and [^18^F]FES [12]. The study was approved the independent ethics committee of the foundation “evaluation of ethics in biomedical research” (CCMO code: NL49312.056.14) and was performed in accordance with standards for Good Clinical Practice, in full compliance with the principles of the 1964 Declaration of Helsinki. Seven healthy post-menopausal women (age 61.5 ± 9.7) were included in the study. Informed consent was obtained from all individual participants included in the study. Exclusion criteria were the use of any concomitant medication, smoking or any other substance dependence. At baseline, a 3D T1-weighted MRI and a dynamic [^18^F]FES PET scan were acquired. Subjects were treated daily with an oral dose of elacestrant (500 mg) for 7 days, to reach steady-state levels in plasma, without hormonal replacement therapy. In 4 subjects, [^18^F]FES PET was repeated 4 h after the last drug dose.

### MRI Acquisition

A structural 3D T1-weighted MRI sequence (matrix size 256 × 256 × 3, voxel size 0.97 × 0.97 × 20, repetition time 11.12 ms, echo time 4.60 ms) on a 3 Tesla Ingenuity TF system (Philips, Netherlands) was acquired for each subject to be used as individual anatomical reference for spatial normalization and co-registration of the PET scans.

### PET Acquisition

A catheter was placed in a brachial vein for intravenous administration of the tracer, and a cannula was inserted into the radial artery of the opposite wrist for blood sampling. PET/CT images were acquired with a Biograph mCT system (Siemens, Knoxville, USA). After a low-dose CT was acquired, a bolus (8.3 ml) of [^18^F]FES (baseline 199 ± 6 MBq; post-dose 209.0 ± 13 MBq) was intravenously injected (0.5 ml/s) and a 90-min dynamic PET scan of the brain was started. The dynamic PET data were reconstructed using a time-of-flight version of the 3D ordered-subsets-expectation–maximization algorithm (3 iterations, 24 subsets) and corrected for decay, attenuation scatter and random coincidences. List-mode data were reconstructed into 33 temporal frames: 6 × 5 s; 4 × 10 s; 4 × 15 s; 3 × 30 s; 3 × 60 s; 4 × 150 s; 3 × 300 s; 6 × 600 s. The final images had a matrix size of 400 × 400 × 111 and a voxel size of 2.03 × 2.03 × 2 mm.

### Blood Sampling and Processing

The radioactivity concentration in arterial blood was continuously measured during the first 30 min of the scan, using an automatic blood sampling system (Veenstra Instruments, Joure, Netherlands). In addition, seven manual samples were taken at approximately 5, 10, 20, 30, 40, 60 and 90 min after tracer injection for calibration of the automated sampler and metabolite analysis. In each sample, the radioactivity concentration in 250 µl of whole-blood and plasma were measured with an automated gamma-counter (Wizard2480, PerkinElmer, USA). The radioactivity concentration was expressed as standardized uptake values (SUV). SUV values were calculated by dividing the measured radioactivity concentration (kBq/mL) by the ratio of the injected dose (kBq) and body weight (g) of the subject. It was assumed that 1 g equals 1 mL.

For metabolite analysis, 50 μl aliquots of the plasma samples were diluted with 100 μl of acetonitrile and centrifuged (5 min, 15000* g*). A 2.5 µl aliquot of the supernatant, was analyzed by thin-layer chromatography, using a silica gel 60 F254 TLC plate (Merck, Germany) and n-hexane/ethyl acetate (7/3) as the mobile phase. A phosphor storage screen (PerkinElmer, USA) was exposed to the TLC plate for approximately 18 h. The phosphor storage screen was scanned with a Cyclone Imaging System (PerkinElmer, USA) and analyzed with OptiQuant Software version 3.0 to determine the percentage of intact [^18^F]FES in plasma. A one-exponential function was fitted to the metabolite data and was used to generate a metabolite-corrected plasma input function.

### Data Analysis and Image Processing

PET images were co-registered to the corresponding T_1_-weighted MRI scan of the same subject, using PMOD version 4.1 (PMOD Technologies LLC, Zürich, Switzerland). Head motion correction was applied to the PET data, if necessary. MRI scans were spatially normalized to Montreal Neurological institute (MNI) space [13] and the transformation matrix was used to align the co-registered PET images. Volumes-of-interest (VOIs) for individual brain regions were obtained from the Hammers atlas [14]. The 83 available brain structures within the Hammers atlas were aggregated into 15 brain regions, as no differences between left and right were expected and no differences within cortical regions. Regions with expected high ER expression, e.g., thalamus, hippocampus and amygdala, were analyzed as separate structures. VOIs for white matter, grey matter, and the whole brain were segmented from the MRI dataset in SPM12 (Wellcome Trust Center for Neuroimaging, UK) with a probability map threshold of 0.5. A VOI for the pituitary gland was drawn manually for each subject in PET-space, using a 3D iso-contour at 40% of the maximum uptake. TACs for each VOI were generated for kinetic modeling.

### Kinetic Modeling

Pharmacokinetic modeling was performed using the metabolite-corrected plasma TAC as the input function and the TAC of whole-blood for blood volume correction. To improve the accuracy of the fits, frame duration and frame mid-time decay were applied as weighting factors for all evaluations. The one-tissue compartment model (1T2k), irreversible two-tissue compartment model (2T3k) and reversible two-tissue compartment model (2T4k) were assessed for fitting the regional TACs, using a fitted fractional volume of blood (V_B_). The 2T4k model was further explored using the V_B_ as a fixed value of 0.05 [15] and by fixation of the influx-efflux rate constant ratio (K_1_/k_2_) to the K_1_/k_2_ ratio of the whole-brain. In addition, Logan graphical analysis was performed with a starting time (t*) of 20 min.

The Akaike Information Criterion (AIC) was used to select the most appropriate model. The standard error in the parameter estimated by the compartment models was used to determine the reliability of in the total volume of distribution (V_T_) and non-displaceable binding potential (BP_ND_) estimates, using an arbitrary cut-off value of 25%. The accuracy of SUV and Logan graphical analysis derived V_T_ was determined via correlational analyses with the macro-parameters of the optimal compartment model. The change in ER availability by the drug was calculated using the Lassen plot [16].

### Statistical Analysis

All statistical analyses were performed using IBM SPSS Statistics (Version 23, Armonk, NY, USA). Differences in kinetic parameter estimates between the baseline and post-dose scans were assessed by generalized estimated equation, using the main effects “brain region” and “treatment” and the interaction “brain region × treatment”. A Bonferroni post-hoc analysis was used for multiple comparisons correction. Results are reported as mean ± standard deviation (SD) and were considered statistically significant if the null hypothesis was rejected at a probability of 95% (*p* < 0.05). The correlation between parameters from different models were examined by Pearson linear regression analysis.

## Results

### Tracer Kinetics and Metabolism

**Fig. 1:**
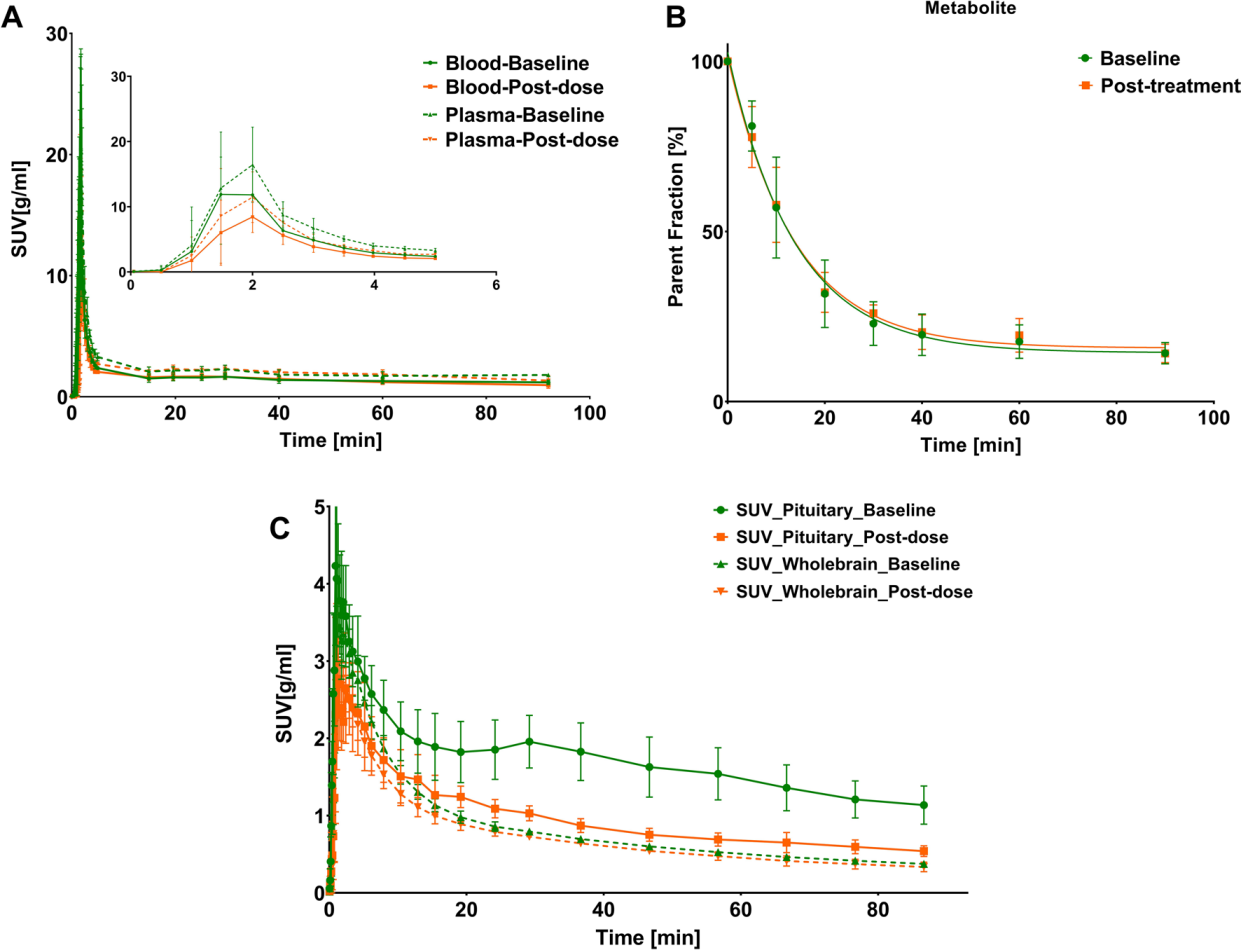
**A** Time-activity curves (SUV) of plasma and whole-blood, **B** parent fraction of [^18^F]FES in plasma, and **C** time-activity curves of the whole brain and pituitary gland from baseline and post-dose scans

Highest [^18^F]FES concentration in blood and plasma was reached after approximately 2 min, which was followed by a rapid decrease (Fig. 1A). Plasma radioactivity comprised 81 ± 7% and 14 ± 3% of intact tracer at 5 and 90 min, respectively (Fig. 1B). No significant effects of drug administration were observed.

[^18^F]FES uptake was relatively homogeneously distributed across different brain regions, although uptake was higher in white matter than in grey matter. Highest brain uptake (SUV 3.47 ± 0.37) was observed at 1.3 ± 0.4 min after tracer administration (Fig. 1C). Peak uptake in the pituitary gland was 4.82 ± 0.61. After the initial peak, the tracer was gradually cleared from the brain, resulting in a SUV of 0.40 ± 0.04 in the whole-brain and 1.17 ± 0.24 in pituitary gland at 80–90 min after tracer injection.

### Compartmental Models

**Fig. 2:**
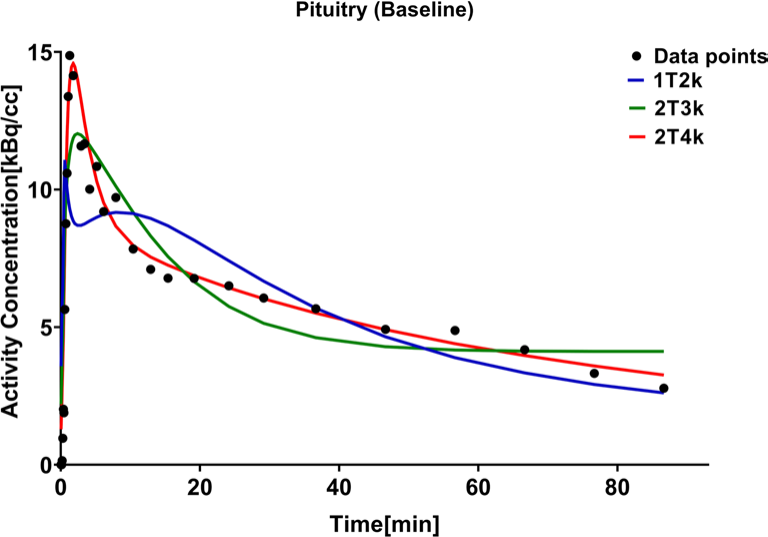
Representative compartment model fits of the measured data points (black dots) in pituitary gland for the 1T2k (blue), 2T3k (green), and 2T4k (red) models at the baseline

The 1T2k model did not fit the data properly (data not shown). At baseline, the 2T4k model fitted the regional [^18^F]FES TACs better than the 2T3k model for the majority of subjects (Fig. 2 and Supplementary Fig. 1). The 2T4k model could not always estimate the outcome parameters with high precision, as 9.0% of V_T_ and 31.6% of BP_ND_ values were estimated with a standard error > 25% (Table 1). To increase the precision and robustness of the estimated parameters, the K_1_/k_2_ of individual brain regions was fixed to the K_1_/k_2_ value of the whole brain and the V_B_ was fixed to 0.05. The K_1_/k_2_ values (Supplementary Table 1) ranged from 0.27 to 1.14 for individual subjects (variance of 0.055) but showed less variance over different brain regions (variance of 0.005 to 0.037 (median 0.010) for individual subjects). Although the pituitary is outside the blood–brain barrier, there were no differences between the K_1_ (*p* = 0.43), k_2_ (*p* = 0.35) and K_1_/k_2_ (*p* = 0.86) values for the whole brain and pituitary and therefore the whole brain K_1_/k_2_ was also used for this brain region. The fixation of the K_1_/k_2_ ratio resulted in lower AIC values, whereas fixation of V_B_ hardly had any effect. Fixation of the K_1_/k_2_ ratio resulted in a reduction of the number of V_T_ estimates at baseline with a standard error > 25% from 9.0% (2T4k) and 6.7% (2T4k-V_B_) to 7.5% (2T4k-K_1_k_2_) and 2.3% (2T4k-K_1_k_2_-V_B_) (Table 1). The fraction of BP_ND_ estimates with a standard error > 25% was reduced from 31.6% (2T4k) and 30.8% (2T4k-V_B_) to 14.3% (2T4k-K_1_k_2_), and 19.5% (2T4k-K_1_k_2_-V_B_).

**Table 1:** The percentage of V_T_ or BP_ND_ estimates with a standard error > 25%, as determined with various modifications of the 2T4k model

	V_T_		BP_ND_	
	Baseline	Post-dose	Baseline	Post-dose
2T4k	9.0%	32.9%	31.6%	89.5%
2T4k-V_B_	6.0%	32.9%	30.8%	85.5%
2T4k-K_1_k_2_	7.5%	14.5%	14.3%	32.9%
2T4k-K_1_k_2_-V_B_	2.3%	11.8%	19.6%	36.8%

**Fig. 3:**
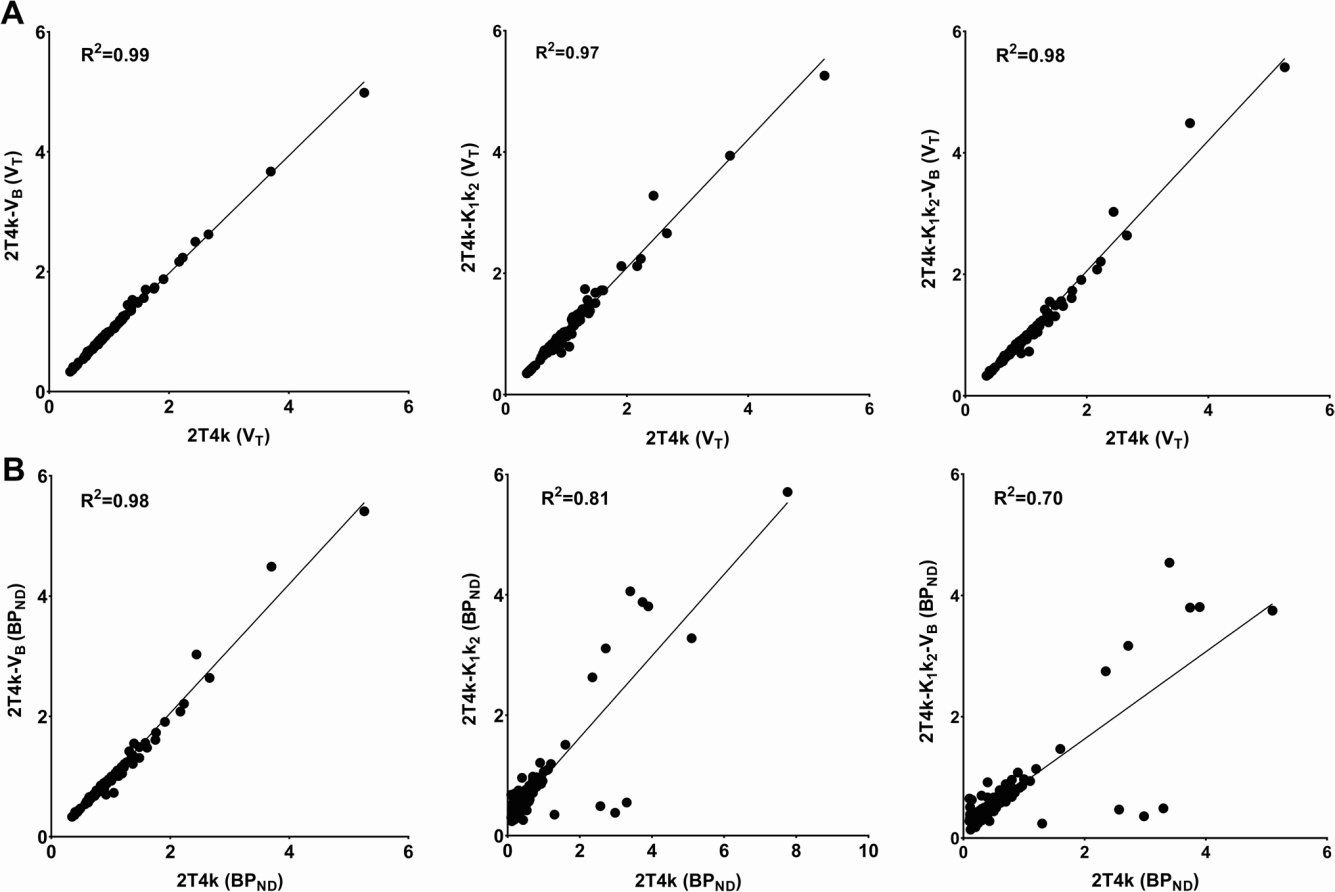
Correlations between V_T_ (**A**) and BP_ND_ (**B**) values estimated with the 2T4k-V_B_, 2T4k-K_1_k_2_, and 2T4k-K_1_k_2_-V_B_ models with the estimates from the 2T4k model. Only V_T_ and BP_ND_ estimates with a standard error < 25% were included in the correlations. Highest values in the plots represent measurements for pituitary gland

Pearson correlations of the models with constrained parameters with the prime compartmental model (2T4k) were performed to assess the bias introduced by fixation of K_1_/k_2_ and V_B_ (Fig. 3). The V_T_ values estimated by the 2T4k model correlated well with the V_T_ estimates from the 2T4k-V_B_ (R^2^ = 0.99, *p* < 0.0001), 2T4k-K_1_k_2_ (R^2^ = 0.97, *p* < 0.0001) and 2T4k-K_1_k_2_-V_B_ model (R^2^ = 0.98, *p* < 0.0001). Worse correlations (R^2^) were observed when BP_ND_ estimates from the 2T4k model were correlated with BP_ND_ estimates derived from the 2T4k-V_B_ (R^2^ = 0.98, *p* < 0.0001), 2T4k-K_1_k_2_ (R^2^ = 0.81, *p* < 0.0001) and 2T4k-K_1_k_2_-V_B_ model (R^2^ = 0.72, *p* < 0.0001), even if only values with a standard error < 25% were included.

Considering the AIC, standard error of the estimated parameters, bias and correlation of outcome parameters with cardinal compartmental model, the 2T4k-K_1_k_2_ model seems to be the preferred model with V_T_ as the preferred outcome parameter.

### Logan Graphical Analysis

**Fig. 4:**
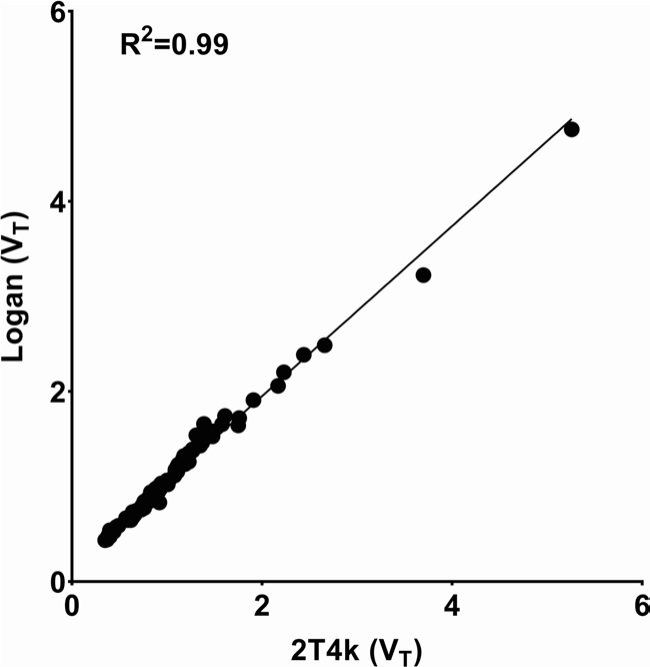
Regression analysis of the distribution volume (V_T_) of [^18^F]FES in individual brain regions, estimated with the 2T4k model and Logan graphical analysis. Only V_T_ estimates with a standard error < 25% were included in the correlations.

Logan graphical analysis could fit the data well. All V_T_ values estimated with Logan graphical analysis had a standard error < 25%. Baseline V_T_ values derived from Logan graphical analysis were strongly correlated with V_T_ values estimated from the 2T4k compartment model (R^2^ = 0.99, *p* < 0.0001; Fig. 4) and the 2T4k-K_1_k_2_ model (R^2^ = 0.98, *p* < 0.0001; Supplementary Fig. 2A), although some underestimation was observed.

**Fig. 5:**
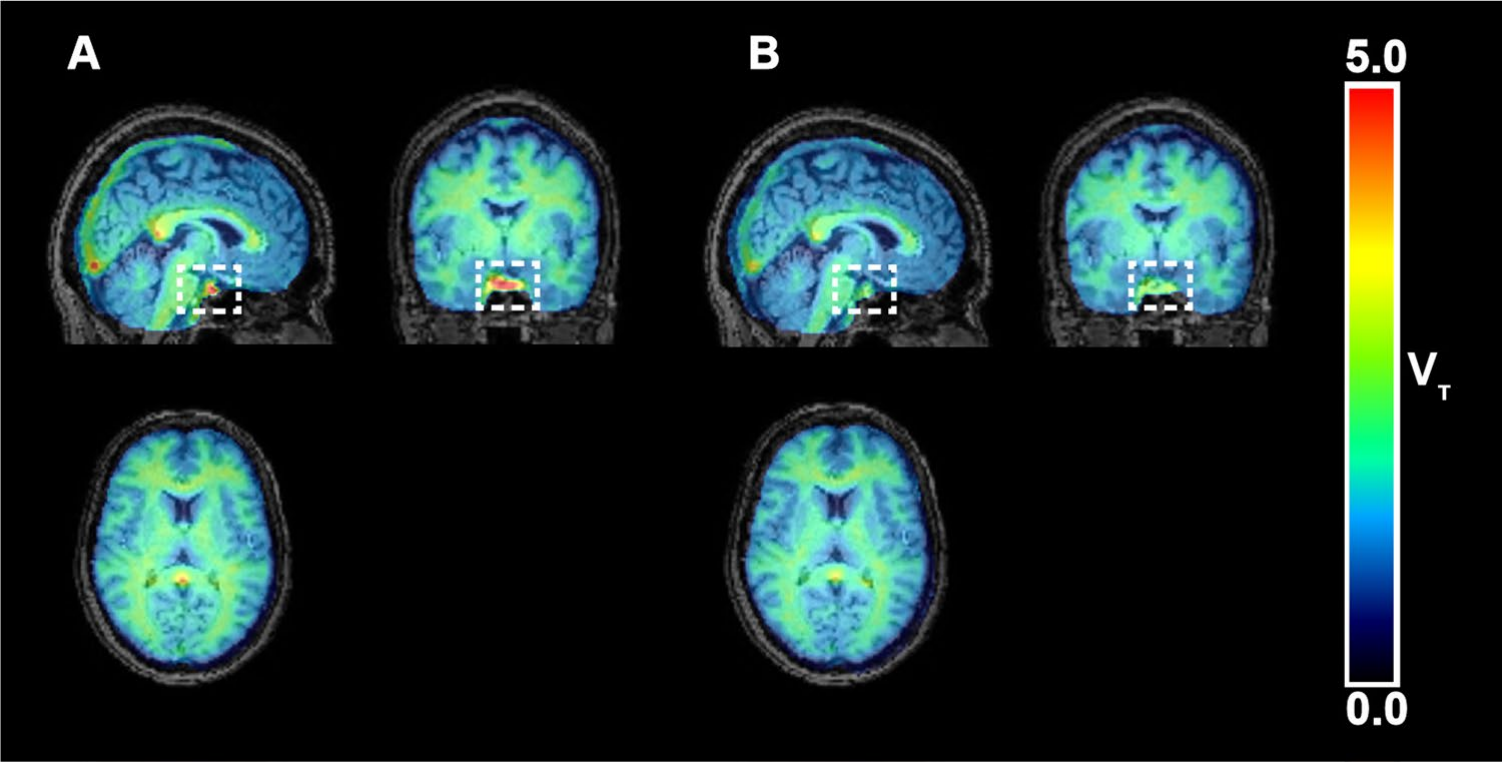
Representative parametric V_T_ images of [^18^F]FES derived from Logan graphical analysis for one subject at baseline (**A**) and post-dose (**B**). Pituitary gland is marked with a dashed square to illustrate the effect of the administrated drug

Representative baseline and post-dose V_T_ images derived from Logan graphical analysis are shown in Fig. 5.

### Standardized Uptake Value

SUV_80-90_ values were only moderately correlated with V_T_ values derived for the 2T4k (R^2^ = 0.61, *p* < 0.0001; Supplementary Fig. 3) or 2T4k-K_1_k_2_ model (R^2^ = 0.58, *p* < 0.0001; Supplementary Fig. 2B). Surprisingly, SUV_80-90_ measurements correlated better with BP_ND_ values estimated from the 2T4k-K_1_k_2_ model (R^2^ = 0.90, *p* < 0.0001; Supplementary Fig. 2C).

### Effect of the Drug

The administration of a nonsteroidal selective ER degrader (elacestrant) before the post-dose [^18^F]FES PET scan reduced the preference for the 2T4k model. The number of subjects and the percentage of brain regions, in which the 2T3k model was preferred based on the AIC values, was similar to those in which the 2T4k model was preferred (Supplementary Fig. 1). Imposing constraints on V_B_ did not substantially improve the robustness of the post-dose outcome parameters (Table 1). However, fixation of the K_1_/k_2_ ratio reduced the percentage of estimated V_T_ values with a standard error > 25% from 32.9% (2T4k) and 32.9%, (2T4k-V_B_) to 14.5% (2T4k-K_1_k_2_) and 11.8% (2T4k-K_1_k_2_-V_B_). The percentage of estimated BP_ND_ values from post-dose scans with a standard error > 25% was reduced from 89.5% and 85.5% to 32.3% and 36.9%, respectively. The V_T_ values derived from the post-dose scans using the 2T4k-K_1_k_2_ model were positively correlated with the V_T_ values assessed with the 2T4k compartmental model (R^2^ = 0.74, *p* < 0.0001). In contrast, the BP_ND_ values estimated with these models did not show any correlation. Therefore, the 2T4k-K_1_k_2_ model with the volume of blood (V_B_) as a fit parameter was considered the optimal model with the V_T_ as the optimal outcome parameter for both the post-dose and the baseline scans.

The drug administration only resulted in a statistically significant reduction of [^18^F]FES uptake in pituitary gland, irrespective whether the V_T_ values derived from the 2T4k-K_1_k_2_ model or Logan analysis, the BP_ND_ from the 2T4k-K_1_k_2_ model, or the SUV_80-90_ were used as outcome parameter (Table 2). Similar results were found when only the four subjects that completed both PET scans were included in the statistical analysis (data not shown). Based on the V_T_ data these subjects, a daily oral dose (500 mg) of elacestrant for 7 days would result in a 64% (V_T_ from 2T4k-K_1_k_2_) or 62% (V_T_ from Logan analysis) reduction in ER availability in the pituitary gland 4 h after the last dose.

**Table 2:** V_T_ and BP_ND_ values (mean ± SD) estimated with the 2T4k-K_1_k_2_ compartment model or Logan graphical analysis and the SUV at 80–90 min after tracer injection at baseline and post-dose for various brain regions

Regions	2T4k-K_1_k_2_ (V_T_)		2T4k-K_1_k_2_ (BP_ND_)		Logan V_T_ (t^*^ = 20)		SUV_80-90_	
	Baseline (*n* = 7)	Post-dose (*n* = 4)	Baseline (*n* = 7)	Post-dose (*n* = 4)	Baseline (*n* = 7)	Post-dose (*n* = 4)	Baseline (*n* = 7)	Post-dose (*n* = 4)
Whole Brain	1.14 ± 0.57	1.36 ± 0.36	0.77 ± 0.20	0.81 ± 0.50	1.09 ± 0.39	1.21 ± 0.27	0.40 ± 0.04	0.38 ± 0.16
Grey Matter	1.08 ± 0.53	1.29 ± 0.33	0.67 ± 0.19	0.71 ± 0.45	1.04 ± 0.37	1.16 ± 0.25	0.37 ± 0.03	0.35 ± 0.15
White Matter	1.25 ± 0.51	1.47 ± 0.36	1.01 ± 0.26	0.94 ± 0.43	1.23 ± 0.43	1.36 ± 0.31	0.47 ± 0.05	0.44 ± 0.19
Brainstem	1.00 ± 0.41	1.15 ± 0.28	0.60 ± 0.18	0.52 ± 0.31	1.05 ± 0.40	1.17 ± 0.24	0.34 ± 0.03	0.34 ± 0.14
Cerebellum	0.83 ± 0.36	1.06 ± 0.25	0.32 ± 0.16	0.41 ± 0.32	0.90 ± 0.35	1.01 ± 0.19	0.27 ± 0.02	0.28 ± 0.11
Thalamus	0.96 ± 0.40	1.06 ± 0.22	0.52 ± 0.14	0.40 ± 0.20	1.00 ± 0.35	1.11 ± 0.23	0.30 ± 0.03	0.28 ± 0.12
Caudate Nucl	1.04 ± 0.78	1.13 ± 0.28	0.51 ± 0.37	0.46 ± 0.35	0.91 ± 0.36	1.03 ± 0.23	0.30 ± 0.04	0.30 ± 0.12
Lentiform Nucl	1.04 ± 0.41	1.19 ± 0.27	0.67 ± 0.17	0.57 ± 0.21	1.12 ± 0.40	1.25 ± 0.23	0.33 ± 0.03	0.32 ± 0.13
Nucl Accumb	0.87 ± 0.33	1.03 ± 0.22	0.39 ± 0.17	0.37 ± 0.22	0.91 ± 0.33	1.02 ± 0.20	0.27 ± 0.04	0.26 ± 0.11
Insula	0.92 ± 0.35	1.09 ± 0.24	0.48 ± 0.20	0.44 ± 0.25	0.99 ± 0.35	1.13 ± 0.22	0.31 ± 0.03	0.31 ± 0.13
Occipital lobe	1.22 ± 0.98	1.45 ± 0.59	0.75 ± 0.46	0.95 ± 0.93	1.01 ± 0.37	1.15 ± 0.24	0.36 ± 0.02	0.35 ± 0.15
Parietal lobe	0.82 ± 0.30	1.17 ± 0.39	0.50 ± 0.20	0.47 ± 0.29	0.93 ± 0.33	1.05 ± 0.27	0.32 ± 0.03	0.31 ± 0.14
Hippocampus	0.97 ± 0.44	1.15 ± 0.24	0.53 ± 0.21	0.53 ± 0.32	0.98 ± 0.35	1.10 ± 0.22	0.33 ± 0.04	0.31 ± 0.13
Amygdala	0.89 ± 0.38	1.03 ± 0.20	0.41 ± 0.17	0.36 ± 0.24	0.93 ± 0.34	1.03 ± 0.18	0.29 ± 0.03	0.28 ± 0.11
Temporal lobe	0.70 ± 0.29	1.18 ± 0.27*	0.51 ± 0.19	0.58 ± 0.41	0.97 ± 0.35	1.08 ± 0.21	0.33 ± 0.02	0.31 ± 0.13
Cingulate gyri	0.85 ± 0.31	1.28 ± 0.36*	0.57 ± 0.20	0.69 ± 0.47	1.01 ± 0.37	1.14 ± 0.25	0.36 ± 0.02	0.35 ± 0.15
FL OFC	1.07 ± 0.64	1.08 ± 0.17	0.62 ± 0.31	0.44 ± 0.23	1.00 ± 0.39	1.09 ± 0.19	0.33 ± 0.05	0.30 ± 0.11
Frontal lobe	0.83 ± 0.29	1.39 ± 0.57*	0.52 ± 0.18	0.86 ± 0.88	0.97 ± 0.35	1.10 ± 0.24	0.33 ± 0.03	0.32 ± 0.14
Pituitary gland	2.98 ± 1.30	1.65 ± 0.59*	3.78 ± 0.99	1.08 ± 0.08*	2.68 ± 1.03	1.68 ± 0.44*	1.17 ± 0.24	0.60 ± 0.18*

Significant differences between the baseline and post-dose scan are indicated with an asterisk: *P < 0.05

Abbreviations: *Caudate Nucl* Caudate nucleus; *Lentiform Nucl* Lentiform nucleus; *Nucl Accumb* Nucleus accumbens; *Cingulate gyri* Cingulate gyrus; *FL OFC* Orbitofrontal cortex and frontal lobe

## Discussion

The primary objectives of this study were to investigate the feasibility of using [^18^F]FES PET for imaging of ER in the human brain, to determine an optimal quantitation approach and to assess the reduction of ER availability after administration of an experimental drug. The 2T4k model was favored over the 1T2k and 2T3k model based on visual assessment of the fit, AIC values and the standard error of the estimated outcome parameters. The 2T4k model provides more robust estimations of the V_T_ than the BP_ND_ and therefore V_T_ is the preferred outcome parameter.

Even with the preferred 2T4k model for quantitation of [^18^F]FES PET, relatively high standard errors, in particular in the estimated BP_ND_, were observed. Fixing the ratio of K_1_/K_2_ enhanced the quality of the fits and decreased the frequency of BP_ND_ estimates with a standard error > 25% by approximately two-fold. Although fixation of the K_1_/K_2_ ratio hardly had any effect on the baseline V_T_ estimates, it did improve the V_T_ estimates from the post-dose scans. The good correlations between the parameter estimated with the 2T4k-K_1_k_2_ model and the 2T4k model and the slope of these correlations being close to one imply that fixing of the K_1_/k_2_ ratio did not introduce any significant bias in the data analysis. Imposing an additional constraint by fixating the V_B_ to 0.05 increased rather than reduced the standard error in the outcome estimates. A plausible explanation could be that the regional blood flow and blood volume differ between various regions of the human brain [15, 17]. Some brain regions, such as cerebellum, have a blood volume that is smaller than 5%, while regions like frontal-lobe may have a higher blood volume fraction.

Logan graphical analysis could fit data well, confirming the findings from compartment modeling that [^18^F]FES acts as a reversible PET tracer in the brain. The strong correlation of V_T_ values estimated with Logan graphical analysis with V_T_ values derived from the 2T4k and 2T4k-K_1_k_2_ model indicate that Logan graphical analysis could be a good alternative for compartment modeling, as it can more reliably estimate V_T_ values. Yet, Logan graphical analysis may introduce some bias due to underestimation of V_T_ or increase of the noise, as fit parameters would be acquired through nonlinear estimation [18]. The SUV_80-90_ only moderately correlated with V_T_ values derived from the compartmental model and therefore is not a good measure for [^18^F]FES binding in the brain.

[^18^F]FES displayed relatively high uptake in white matter, whereas uptake was relatively low and homogeneously distributed in grey matter regions. The relatively high uptake observed in white matter was not reduced after the administration of the drug, indicating that the uptake in white matter is mainly due to non-specific binding. This can be explained by the relatively high lipophilicity of the PET tracer. Low estrogen receptor expression in white matter was found in autoradiography studies in the female rat and monkey, supporting the lack of specific binding [19, 20]. Administration of elacestrant did not have any effect on the blood kinetics of the tracer or its metabolism but reduced the preference for the 2T4k model in post-dose scans, resulting in an approximately equal number of brain regions in which the 2T3k model was preferred. One reason for this shift in model preference could be that the post-dose scans are less influenced by the second compartment, representing specific binding, due to saturation of the receptor. While in case of complete receptor saturation the 1T2k model would be expected to give a better fit, we did not find this. Apparently, there is still binding of [^18^F]FES so that the fit is better when the rate constants k_3_ and k_4_ are estimated as well. As the signal to noise ratio of PET data in areas with low receptor availability is intrinsically low, it is more difficult to accurately estimate the rate constants, in particular k_3_ and k_4_, which could result in the preference for 2T3k.

We could only demonstrate specific binding in pituitary gland, which is in line with findings from animal studies [7, 21]. Administration of elacestrant led to a reduction of ER availability of 62–64% in the pituitary gland. Besides pituitary gland, no statistically significant effect of the drug on [^18^F]FES binding could be observed in any brain region. This could be due to the relatively low ER density the brain. ER-mediated binding may thus have been obscured by high levels of non-specific binding. Previous studies in rats reported specific [^18^F]FES binding in hypothalamus [7]. Since the hypothalamus in rats is located close to the pituitary gland, this apparent specific binding might be the result of spillover from the pituitary gland. Another reason why specific binding was only observed in the pituitary gland could be that the pituitary gland is located outside the blood–brain barrier (BBB). [^18^F]FES or elacestrant might be a substrate for an efflux pump in the BBB and be extruded from the brain before it can reach cerebral ER. Based on the TACs of [^18^F]FES uptake in the brain, however, it appears that BBB penetration is not impaired, since peak uptake in the whole brain is in the same range as peak uptake in the pituitary gland (SUV 3.5 vs. 4.8). In an intracranial mouse model elacestrant levels in the tumor were comparable to levels observed in plasma, suggesting that elacestrant can cross the BBB [11]. Furthermore, in a clinical study elacestrant was detected in cerebrospinal fluid after oral administration [10]. Finally, it has to be mentioned that the low number of subjects is a limitation of the study. Especially the lack of significant specific binding in other regions than the pituitary gland could be due to the small number of subjects that underwent both the baseline and post-dose scan, which limits the statistical power of the study.

Due to the poor sensitivity to detect specific binding in the brain, [^18^F]FES does not seem to be a suitable radioligand for measuring the cerebral ER expression in patients with psychiatric or neurological diseases. However, [^18^F]FES PET could be useful for investigation of the role of ER in the pituitary gland in stress-related disorders. Stress activates the hypothalamic–pituitary–adrenal (HPA) axis. Several studies have shown that the response of the HPA-axis to stress can be modulated by ER-mediated signaling and that fluctuations in circulating estrogen levels affect the activity of the HPA-axis [22, 23]. The role of ER in the pituitary gland largely remains to be elucidated and [^18^F]FES PET could provide a useful tool for this purpose.

## Conclusion

Our study indicates that the reversible 2T4k-K_1_k_2_ model is the model of choice to describe the pharmacokinetics of [^18^F]FES in human brain. Besides the compartment model, Logan graphical analysis can also be applied as a robust approach for quantification of [^18^F]FES uptake in the human brain. [^18^F]FES showed ER-mediated uptake in pituitary, but not in the brain. Therefore, [^18^F]FES only seems to be a suitable PET tracer for quantification of ER expression in tissues with high ER density like pituitary gland.

### Supplementary Information

Below is the link to the electronic supplementary material.

Supplementary file1 (DOCX 234 kb)
