# Supplementary material for: Pharmacokinetic Analysis of [18F]FES PET in the Human Brain and Pituitary Gland
Source: Mol Imaging Biol. 2024 Jan 23;26(2):351–9. doi: 10.1007/s11307-023-01880-z (PMC10972926; doi:10.1007/s11307-023-01880-z)
Supplement: Supplementary file 1 — Supplementary file1 (DOCX 234 kb) [file 11307_2023_1880_MOESM1_ESM.docx]

**Electronic Supplementary Material**

**Pharmacokinetic Analysis of [^18^F]FES PET in the Human Brain and Pituitary Gland**

**Journal: Molecular Imaging and Biology**

Nafiseh Ghazanfari, Janine Doorduin, Chris W.J. van der Weijden, Antoon. T.M. Willemsen,

Andor W.J.M. Glaudemans, Aren van Waarde, Rudi A.J.O. Dierckx, Erik F.J. de Vries

*University of Groningen, University Medical Center Groningen,*

*Department of Nuclear Medicine and Molecular Imaging,*

*Hanzeplein 1, 9713GZ, Groningen, The Netherlands*

Corresponding author:

Janine Doorduin, PhD

E-Mail: j.doorduin@umcg.nl

Tel: +31-50-3613541

**Supplementary figure 1.** Model preference. **(a)** The number of subjects and **(b)** the percentage of brain regions, for which a particular compartment model gave the lowest AIC value.


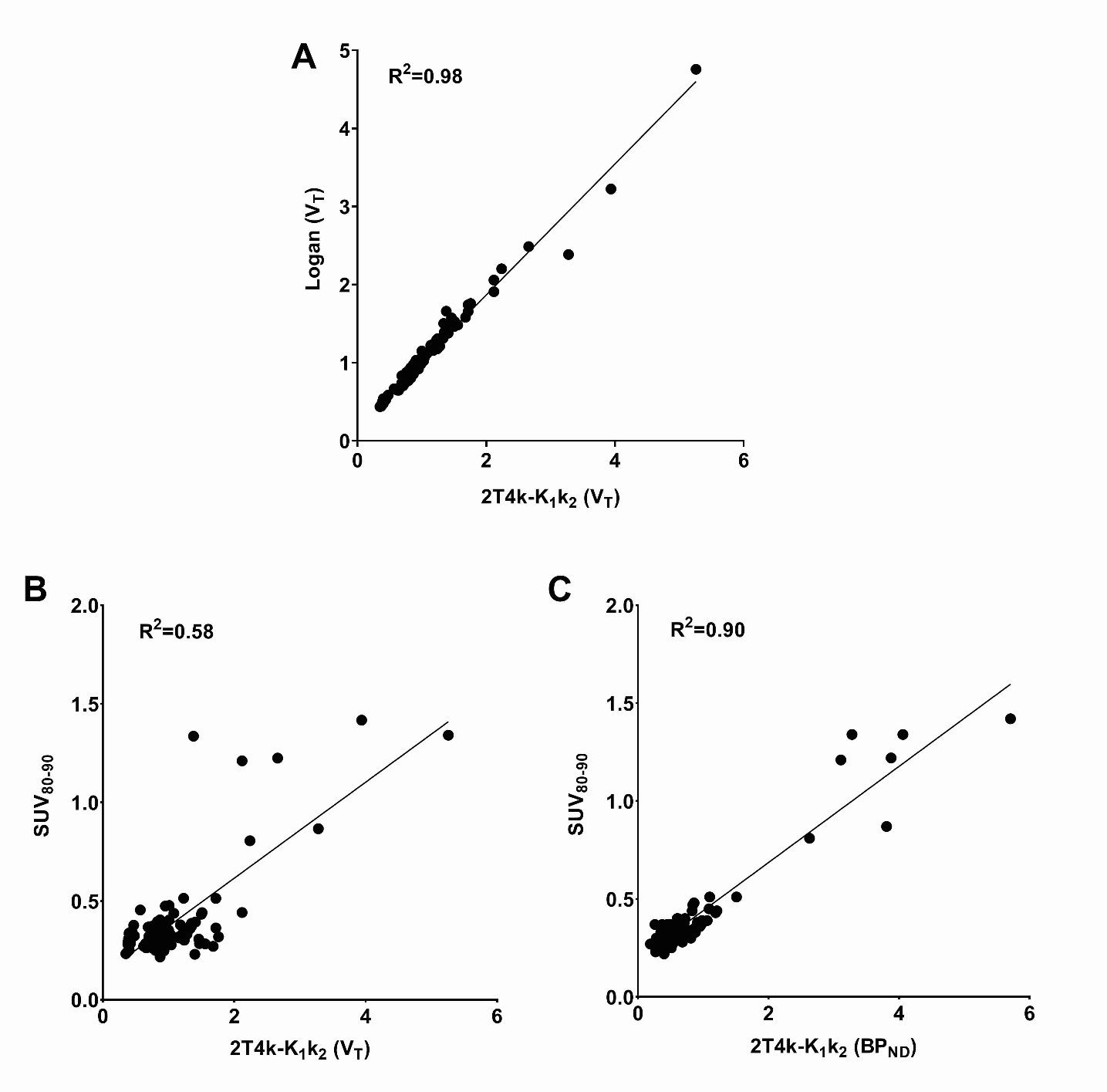


**Supplementary figure 2.** Linear Pearson correlation of the distribution volume (V_T_) of [^18^F]FES in individual regions of the human brain, estimated with the 2T4k-K_1_k_2_ model and Logan graphical analysis (**a**), and of regional SUV values between 80 and 90 min after [^18^F]FES injection (SUV_80-90_) with V_T_ and BP_ND_ values derived from the 2T4k-K_1_k_2_ compartment model (**b, c**). Only V_T_ and BP_ND_ estimate with a standard error of < 25% were included in the correlations.


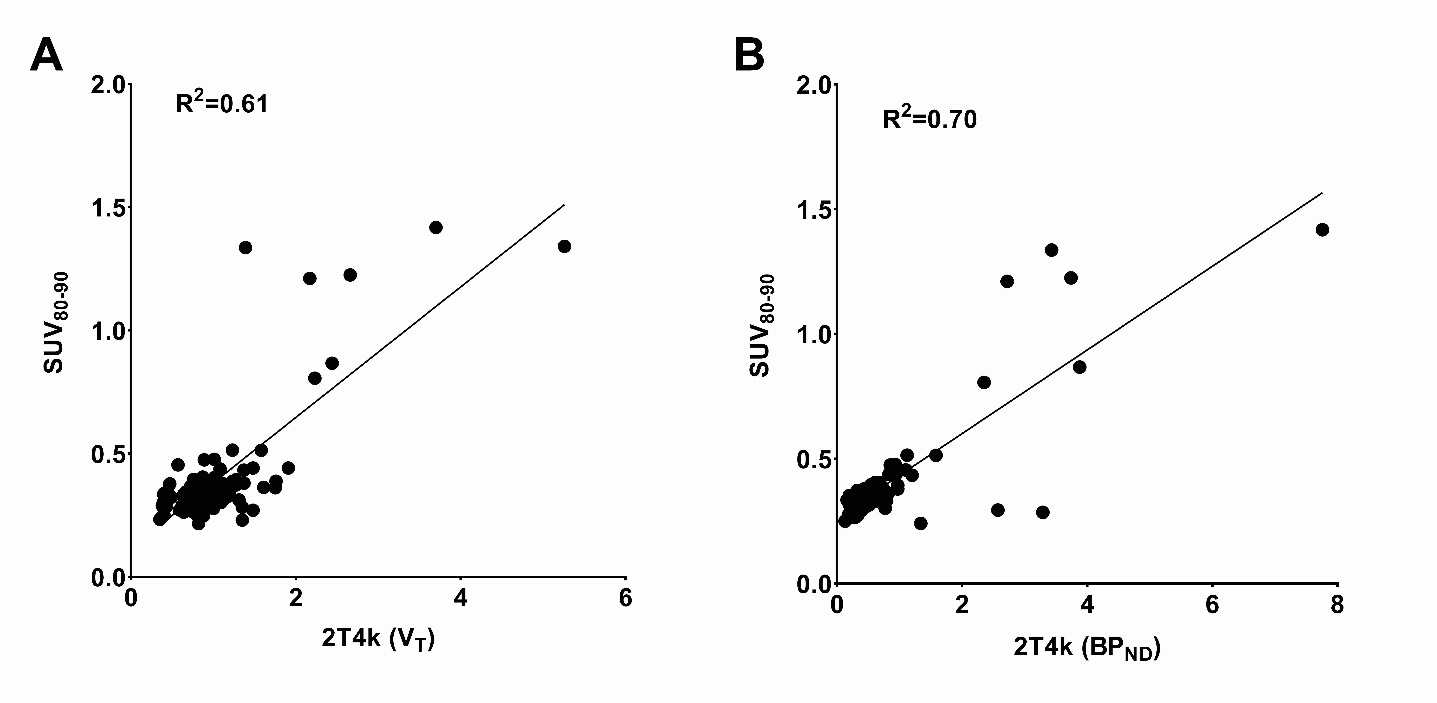


**Supplementary figure 3.** Linear Pearson correlation of regional SUV values between 80 and 90 after [^18^F]FES injection (SUV_80-90_) with V_T_ (**a**) and BP_ND_ values derived from the 2T4k compartment model (**b**). Only V_T_ and BP_ND_ estimate with a standard error of < 25% were included in the correlations.

**Supplementary table 1:** K_1_, k_2_ and K_1_/k_2_ values (mean ± SD) estimated with the 2T4k compartment model at baseline and post-dose for various brain regions.

| **Regions** |  | **K_1_** | | | | | |  | **k_2_** | | | | | |  | **K_1_/k_2_** | | | | | |
| --- | --- | --- | --- | --- | --- | --- | --- | --- | --- | --- | --- | --- | --- | --- | --- | --- | --- | --- | --- | --- | --- |
|  |  | **Baseline (n=7)** | | | **Post-dose (n=4)** | | |  | **Baseline (n=7)** | | | **Post-dose (n=4)** | | |  | **Baseline (n=7)** | | | **Post-dose (n=4)** | | |
| Whole Brain |  | 0.24 | ± | 0.18 | 0.35 | ± | 0.23 |  | 0.36 | ± | 0.10 | 0.45 | ± | 0.18 |  | 0.61 | ± | 0.24 | 0.74 | ± | 0.19 |
| Grey Matter |  | 0.25 | ± | 0.19 | 0.49 | ± | 0.28 |  | 0.37 | ± | 0.10 | 1.34 | ± | 1.57 |  | 0.63 | ± | 0.25 | 0.68 | ± | 0.32 |
| White Matter |  | 0.21 | ± | 0.16 | 0.32 | ± | 0.22 |  | 0.33 | ± | 0.10 | 0.43 | ± | 0.19 |  | 0.59 | ± | 0.23 | 0.71 | ± | 0.19 |
| Brainstem |  | 0.24 | ± | 0.22 | 0.52 | ± | 0.38 |  | 0.29 | ± | 0.10 | 1.08 | ± | 0.92 |  | 0.71 | ± | 0.31 | 0.67 | ± | 0.26 |
| Cerebellum |  | 0.32 | ± | 0.24 | 0.58 | ± | 0.36 |  | 0.57 | ± | 0.29 | 1.17 | ± | 0.99 |  | 0.64 | ± | 0.35 | 0.69 | ± | 0.28 |
| Thalamus |  | 0.32 | ± | 0.15 | 0.58 | ± | 0.43 |  | 0.97 | ± | 0.77 | 1.07 | ± | 0.81 |  | 0.53 | ± | 0.30 | 0.69 | ± | 0.25 |
| Caudate Nucl |  | 0.30 | ± | 0.21 | 0.70 | ± | 0.44 |  | 0.42 | ± | 0.09 | 2.58 | ± | 3.17 |  | 0.67 | ± | 0.27 | 0.57 | ± | 0.25 |
| Lentiform Nucl |  | 0.35 | ± | 0.25 | 0.63 | ± | 0.38 |  | 0.38 | ± | 0.10 | 0.90 | ± | 0.66 |  | 0.90 | ± | 0.36 | 0.88 | ± | 0.30 |
| Nucl Accumb |  | 0.27 | ± | 0.15 | 0.47 | ± | 0.31 |  | 0.75 | ± | 0.67 | 0.85 | ± | 0.62 |  | 0.55 | ± | 0.27 | 0.67 | ± | 0.19 |
| Insula |  | 0.27 | ± | 0.21 | 0.68 | ± | 0.45 |  | 0.36 | ± | 0.09 | 1.82 | ± | 2.02 |  | 0.71 | ± | 0.30 | 0.66 | ± | 0.27 |
| Occipital lobe |  | 0.29 | ± | 0.22 | 0.56 | ± | 0.32 |  | 0.40 | ± | 0.12 | 1.67 | ± | 2.11 |  | 0.66 | ± | 0.26 | 0.71 | ± | 0.35 |
| Parietal lobe |  | 0.27 | ± | 0.19 | 0.55 | ± | 0.33 |  | 0.40 | ± | 0.11 | 1.82 | ± | 2.34 |  | 0.62 | ± | 0.23 | 0.66 | ± | 0.32 |
| Hippocampus |  | 0.21 | ± | 0.15 | 0.57 | ± | 0.40 |  | 0.29 | ± | 0.08 | 1.54 | ± | 1.56 |  | 0.66 | ± | 0.25 | 0.58 | ± | 0.22 |
| Amygdala |  | 0.21 | ± | 0.13 | 0.53 | ± | 0.36 |  | 0.49 | ± | 0.61 | 1.92 | ± | 2.04 |  | 0.68 | ± | 0.34 | 0.57 | ± | 0.28 |
| Temporal lobe |  | 0.25 | ± | 0.19 | 0.62 | ± | 0.40 |  | 0.35 | ± | 0.10 | 1.85 | ± | 2.12 |  | 0.67 | ± | 0.25 | 0.61 | ± | 0.25 |
| Cingulate gyri |  | 0.32 | ± | 0.26 | 0.71 | ± | 0.45 |  | 0.43 | ± | 0.13 | 2.52 | ± | 3.19 |  | 0.67 | ± | 0.27 | 0.63 | ± | 0.30 |
| FL OFC |  | 0.29 | ± | 0.21 | 0.76 | ± | 0.53 |  | 0.42 | ± | 0.10 | 2.56 | ± | 3.19 |  | 0.66 | ± | 0.28 | 0.62 | ± | 0.26 |
| Frontal lobe |  | 0.29 | ± | 0.22 | 0.68 | ± | 0.45 |  | 0.42 | ± | 0.11 | 2.33 | ± | 2.99 |  | 0.65 | ± | 0.25 | 0.62 | ± | 0.27 |
| Pituitary gland |  | 0.27 | ± | 0.21 | 0.77 | ± | 0.98 |  | 0.43 | ± | 0.19 | 1.07 | ± | 1.35 |  | 0.56 | ± | 0.16 | 0.78 | ± | 0.14 |

Abbreviations: Caudate nucleus (Caudate Nucl), Lentiform nucleus (Lentiform Nucl), nucleus accumbens (Nucl Accumb), Cingulate gyrus (Cingulate gyri), orbitofrontal cortex and frontal lobe (FL OFC)
